# A mutation in the major autophagy gene, *WIPI2*, associated with global developmental abnormalities

**DOI:** 10.1093/brain/awz075

**Published:** 2019-04-10

**Authors:** Musharraf Jelani, Hannah C. Dooley, Andrea Gubas, Hussein Sheikh Ali Mohamoud, Muhammad Tariq Masood Khan, Zahir Ali, Changsoo Kang, Fazal Rahim, Amin Jan, Nirmal Vadgama, Muhammad Ismail Khan, Jumana Yousuf Al-Aama, Asifullah Khan, Sharon A Tooze, Jamal Nasir

**Affiliations:** 1 Department of Genetic Medicine, Faculty of Medicine, King Abdulaziz University, Jeddah, Saudi Arabia; 2 Centre for Omic Sciences, Islamia College Peshawar, Pakistan; 3 The Francis Crick Institute, Molecular Cell Biology of Autophagy, London, UK; 4 SW Thames Regional Genetics Laboratory, St. George’s University Hospitals NHS Foundation Trust, UK; 5 North West School of Medicine, Peshawar, Pakistan; 6 Laboratory for Genome Engineering, Division of Biological Sciences, King Abdullah University of Science and Technology, Thuwal 23955–6900, Saudi Arabia; 7 Department of Biology and Institute of Basic Sciences, Sungshin Women’s University, Seoul, Republic of Korea; 8 Department of Physiology, Bacha Khan Medical College, Mardan, Pakistan; 9 Genetics Unit, Cell Biology and Genetics Research Centre, Molecular and Clinical Sciences Research Institute, St. George’s University of London, London, UK; 10 Zoology Department, Islamia College University, Peshawar, Pakistan; 11 Department of Biochemistry, Abdul Wali Khan University Mardan, Pakistan

**Keywords:** autophagy, gene, WIPI2, LC3, exome sequencing

## Abstract

We describe a large consanguineous pedigree from a remote area of Northern Pakistan, with a complex developmental disorder associated with wide-ranging symptoms, including mental retardation, speech and language impairment and other neurological, psychiatric, skeletal and cardiac abnormalities. We initially carried out a genetic study using the HumanCytoSNP-12 v2.1 Illumina gene chip on nine family members and identified a single region of homozygosity shared amongst four affected individuals on chromosome 7p22 (positions 3059377–5478971). We performed whole-exome sequencing on two affected individuals from two separate branches of the extended pedigree and identified a novel nonsynonymous homozygous mutation in exon 9 of the *WIPI2 *(WD-repeat protein interacting with phosphoinositide 2) gene at position 5265458 (c.G745A;pV249M). WIPI2 plays a critical role in autophagy, an evolutionary conserved cellular pathway implicated in a growing number of medical conditions. The mutation is situated in a highly conserved and critically important region of WIPI2, responsible for binding PI(3)P and PI(3,5)P2, an essential requirement for autophagy to proceed. The mutation is absent in all public databases, is predicted to be damaging and segregates with the disease phenotype. We performed functional studies *in vitro *to determine the potential effects of the mutation on downstream pathways leading to autophagosome assembly. Binding of the V231M mutant of WIPI2b to ATG16L1 (as well as ATG5–12) is significantly reduced in GFP pull-down experiments, and fibroblasts derived from the patients show reduced WIPI2 puncta, reduced LC3 lipidation and reduced autophagic flux.

## Teaser

Defects in autophagy are implicated in a growing number of diseases. Jelani *et al.* identify a mutation in *WIPI2*, a major autophagy gene, associated with a multisystemic global developmental disorder. Functional studies in cell lines derived from patients reveal significant reductions in the classic hallmarks of autophagy.

## Introduction

Autophagy, a self-eating cellular pathway capable of engulfing cytoplasmic contents, is triggered under various conditions, including oxidative stress, starvation, microbial infections, or accumulation of misfolded proteins (Giordano *et al.*, 2013; Green and Levine, 2014; Hurley and Schulman, 2014; Stepp *et al.*, 2014; Yu *et al.*, 2018). In particular, its role in various clinical conditions has attracted interest as a possible therapeutic target for cancer and neurodegenerative conditions, including Huntington’s disease (Choi *et al.*, 2013; Nixon, 2013; Ebrahimi-Fakhari *et al.*, 2016).

This cellular pathway leading to autophagy is highly conserved with more than 40 autophagy related genes (ATG) in yeast, of which about half are conserved in humans. Initiation of autophagy begins with the formation of an autophagosome, which delivers material to the lysosome. *WIPI2*, the mammalian homologue of the yeast *ATG18 *gene, plays a key role in autophagy. It binds the omegasome, a phosphatidylinositol 3-phosphate [PI(3)P] rich domain of the endoplasmic reticulum from which the mature autophagosome develops (Axe *et al.* 2008; Polson *et al.* 2010). Through its interaction with ATG16L1, WIPI2b recruits the ATG12–5–16L1 complex to the phagophore and therefore enables LC3 lipidation at this site and subsequent autophagosome formation (Dooley *et al.*, 2015). LC3 belongs to the ATG8 family of small ubiquitin-like proteins, which become lipidated on the surface of the phagophore and autophagosome. Ultimately, the LC3 proteins and other family members are degraded along with the autophagosome content in the lysosome and then returned to the cytosol. Cells depleted of WIPI2 fail to form autophagosomes and engulfment of pathogenic bacteria, including *Salmonella*, is also severely impaired. Equally, WIPI2b mutants, unable to bind ATG16L1, inhibit LC3 lipidation and LC3 puncta formation (Dooley *et al.*, 2014). Site-directed mutagenesis in yeast and crystallization of the yeast WIPI2 orthologue HSV2 have led to the understanding of how the FRRG motif in HSV2 is capable of binding PI(3)P and phosphatidylinositol 3,5-bisphosphate [PI(3,5)P2]. Within the FRRG motif, two head groups on the PI(3)P are bound in site 1 and site 2, located on blades 5 and 6 respectively of the seven-bladed β-propeller structure (Baskaran *et al.*, 2012; Krick *et al.*, 2012). This mode of binding is likely to be conserved in WIPI2, which has been shown to require PI(3)P to bind membranes via the FRRG motif (Polson *et al.*, 2010).

Evidence linking autophagy to genetic diseases is growing, especially for neurological conditions (Akizu *et al.*, 2015; Ebrahimi-Fakhari *et al.*, 2016;Menzies *et al.*, 2017). Mutations in the familial Parkinson’s disease genes, *PINK1 *and parkin (*PARK2*), involve mitophagy, a form of autophagy involved in the removal of damaged mitochondria (Manzanillo *et al.*, 2013; Behr and Schurr, 2013). Genetic variants in the *PARK2 *gene in humans and mice deficient in parkin, also show increased susceptibility to intracellular pathogens such as salmonella and mycobacteria. A common variant in ATG16L1, identified as a risk factor for Crohn’s disease, is associated with reduced autophagy (Kaser and Blumberg, 2014; Murthy *et al.*, 2014), and hemizygous and heterozygous mutations in *WIPI4 *have been identified in sporadic patients with BPAN (β-propeller associated neurodegeneration)—a neurological condition that is a subtype of NBIA (neurodegeneration with brain iron accumulation), characterized by childhood onset psychomotor delay, iron deposits in the brain and patients becoming bedridden (Haack *et al.*, 2012; Hayflick *et al.* 2013; Saitsu *et al.*, 2013; Seibler *et al.*, 2018).

**Figure 1:**
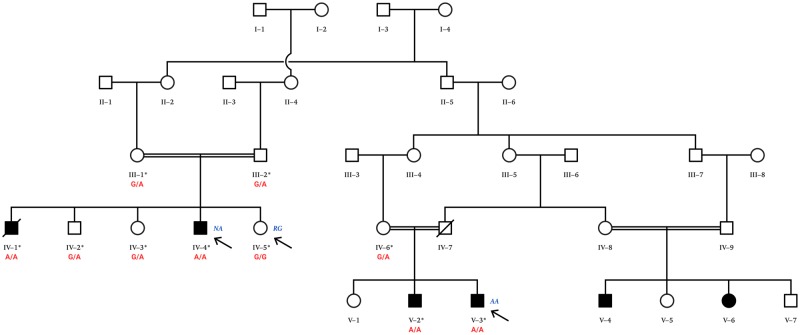
**Large consanguineous pedigree from Northern Pakistan.** Affected members of the family present with a complex developmental disorder affecting multiple tissues segregating as an autosomal recessive condition. Two affected individuals (Patients NA and AA) from two separate branches of the family were selected for exome sequencing. Cells derived from an unaffected healthy sibling (Subject RG) and an affected individual (Patient AA) were used in the functional studies. Individuals NA, RG and AA are indicated with arrows. The cG745A mutation reported in this family was confirmed by Sanger sequencing (Fig. 3) in 10 family members marked with an asterisk. Their corresponding genotypes (G/G, G/A, A/A) are given.

Combining array CGH (comparative genomic hybridization) to identify shared regions of homozygosity in affected family members, with exome sequencing, we previously identified several disease-causing genes associated with rare disorders present in consanguineous pedigrees (Alrayes *et al.*, 2015, 2016; Ahmed *et al.*, 2015; Mohamoud *et al.*, 2018). Here, we report a novel homozygous recessive mutation in the major autophagy gene, *WIPI2*, associated with global developmental abnormalities in a large consanguineous family (Fig. 1). We show that WIPI2 V249M mutation results in reduced WIPI2-positive membranes and a decrease in autophagosome formation and flux.

## Materials and methods

### DNA isolation from peripheral blood

The family belonged to a community living in a remote village in the northern area of Pakistan. Peripheral blood samples from 10 family members, including both sets of parents, were collected in K3 EDTA tubes (BD Vacutainer) and stored at 4°C for genomic DNA isolation and quantification, using commercially available kits (QIAamp, Qiagen) and a Nanodrop-2000 spectrophotometer (Thermo Scientific), respectively.

### Ethical consent

Prior to commencement of the study, informed written consent was obtained from all healthy participants and from the legal guardians of the affected individuals, including agreement on publishing the research outcomes. The study was approved, according to the Declaration of Helsinki, by the Institutional Review Board (IRB) of the Princess Al-Jawhara Albrahim Centre of Excellence in Research of Hereditary Disorders and the Unit of Biomedical Ethics Research Committee (ref. #24–14), Faculty of Medicine, King Abdulaziz University, Jeddah, Saudi Arabia.

### Study subjects

All the affected and unaffected individuals were initially examined by clinicians from The Institute of Basic Medical Sciences, Peshawar, Pakistan and detailed information regarding the disease history of the affected patients and the pedigree structure was obtained by interviewing the patients who were then referred to specialists for detailed cardiac, neurological, orthopaedic, psychiatric, psychological, ophthalmic, radiological and other investigations.

The family tree, shown in Fig. 1, was drawn after interviewing the family elders. Four affected, three unaffected, and three parental samples were available for genetic analysis from two loops of the pedigree (Fig. 1). Peripheral blood samples from the 10 available family members were collected in EDTA tubes and the genomic DNA was extracted using standard methods.

### Clinical histories

In addition to the details reported in the results section, other clinical findings were noted. Dyskinesia and dysarthria are prominent features. The patients lack in higher instincts such as self-actualization and self-esteem. Love/belonging and safety concerns are at minimal. The basic human physiological instincts are intact. Enuresis and nocturia are commonly experienced. Heterogeneity in playful behaviour was noticed among the patients, with some exhibiting a calm behaviour, while others having playful attitude. The memory (both short- and long-term) is impaired. Habituation to oral tobacco (snuff) is found in one, Patient AA. Primitive sexual desires are deranged. Reproductive organs have normal morphological features. Regular nocturnal emissions are reported. The respiratory and gastrointestinal systems do not exhibit any abnormal features. Physical examination, radiological assessment and biochemical findings are suggestive of normal respiratory and gastrointestinal functions. In Patient NA, the haematological indices are suggestive of iron deficiency anaemia. This corresponded well with his poor socio-economic status and inadequate hygiene issues.

### Homozygosity mapping

To investigate the possible involvement of chromosomal aberrations and for the purpose of homozygosity mapping, HumanCytoSNP-12 v2.1 microarray containing 200 000 single nucleotide polymorphisms (SNPs) throughout the genome was used together with iScan platform (Illumina) to analyse all 10 available samples described. The common region of homozygosity among the affected individuals was mapped using GenomeStudio data analysis software (Illumina).

### Whole-exome sequencing and analysis

Whole exome paired-end sequencing was performed by Macrogen starting with 2 μg genomic DNA (260/280 >1.7) by exome capture using the 51 Mb SureSelect All Exon V4 kit (Agilent Technologies) on a HiSeq 2000 platform (Illumina) generating 50 509 146 (Patient AA) and 51 605 788 (Patient NA) reads, yields of 5 101 423 746 and 5 212 184 588 base pairs, respectively, average read length of 101 bp and read depth of 99.7 and 101.8, respectively. Ninety-seven per cent of target regions showed >10× coverage.

For bioinformatic analyses, we used Lasergene Genomic Suite V.12 software package (DNASTAR, Madison, WI, USA). Briefly, FASTQ files were aligned to hg19 (NCBI build GRCh37) using SeqMan NGen 12.2. ArrayStar v.12 was employed to annotate variant alleles based on dbSNP 142. The mapped variants were compared with dbSNP (http://www.ncbi.nlm.nih.gov/snp/) and the 1000 Genomes (http://www.1000genomes.org/) databases. All homozygous variants, including single nucleotide variants shared by the two affected individuals, were filtered using ArrayStar v.12.

Because the family presented with an autosomal recessive mode of inheritance, we filtered candidate variants using the following criteria: (i) homozygous or compound heterozygous in affected individuals, and heterozygous in obligate carriers for the mutant allele; (ii) present in coding exons; (iii) non-synonymous, frame-shift, gain/loss of stop codons; (iv) having a functional effect, e.g. predicted to be pathogenic or deleterious by *in silico* prediction software including Provean (Choi *et al.*, 2012) and MutationTaster2 (Schwarz *et al.*, 2014); (v) present within the region of homozygosity identified in SNP microarray genotyping; and (vi) not present in in-house exome sequence data obtained from 24 normal unrelated Pakistani individuals.

### PCR and gel electrophoresis

*WIPI2 *forward and reverse primers (5′-TTTGAGAAGG TGGCTAGAGG-3′ and 5′-ACTGGCTAACGATGTCAGAG-3′, respectively) were designed using Primer 3 software (Koressaar and Remm, 2007). PCR amplification was performed in a Mastercycler pro Thermal Cycler (Eppendorf) in a 10 μl reaction volume containing 1 μl of genomic DNA (10–30 ng/µl), 0.1 μl of DreamTaq^TM^ DNA Polymerase (Thermo Scientific), 1.0 μl of 10× DreamTaq^TM^ Green Buffer (Thermo Scientific), 0.4 μl of a dNTP mix containing dATP, dTTP, dCTP, and dGTP at a concentration of 5 mM each (Thermo Scientific), 0.4 μl of each primer (Sigma-Aldrich) at a concentration of 5 pmol, and distilled deionized water. The reaction conditions were as follows: 95°C for 3 min, followed by 40 cycles at 94°C for 30 s, 56°C for 30 s, 72°C for 30 s, and a final extension at 72°C for 7 min. PCR products were separated by electrophoresis in 1.2% agarose gels made with Tris-acetate-EDTA buffer (Alpha laboratories).

### DNA sequencing

The candidate variant was confirmed by Sanger sequencing to validate the whole exome sequencing data data. Ten available family members were screened to determine whether or not the variant in question co-segregated with disease status. PCR products were purified following the Exonuclease I (New England Biolabs)–shrimp alkaline phosphatase (Affymetrix) (ExoSAP) protocol for sequencing reactions. The mixtures were incubated at 37°C for 5 min followed by deactivation of the ExoSAP reaction at 95°C for 5 min.

Sequencing was performed bidirectionally using the BigDye® Terminator Chemistry (Sequenase v3.1 Cycle Sequencing Kit; Applied Biosystems). The sequencing reactions were performed in a 10 μl volume containing 1.0 μl of forward or reverse primer (5 pmol), 2.0 μl of the PCR product, 2.0 μl of 5× BigDye® Buffer, 0.5 μl of BigDye® Terminators and 4.5 μl of distilled deionized water. The BigDye XTerminator® Purification Kit (Applied Biosystems) was used to eliminate unincorporated dye terminators and free salts by adding 20 μl of the SAM^TM^ solution and 5 μl of the XTerminator^TM^ to each of the 10 μl sequencing reactions. The mixture was placed on a shaker at full speed for 30 min and then centrifuged at 1000 rpm for 2 min. Samples were run on the 3130xl automated DNA sequencer (Applied Biosystems). Sequencing data were analysed using the CodonCode Aligner v.4.2.4 (CodonCode Corp., USA) and Finch TV (GeoSpiza, Seattle, WA). Wild-type sequence was derived from the Ensembl Genome Browser (Wellcome Trust Sanger Institute, Cambridge, UK).

### Derivation of human fibroblast cell lines from skin biopsies

Skin biopsies were directly placed in 10 ml of transport media [Hams F10 (Sigma) supplemented with amoxicillin (100 µg/ml)] and transported at room temperature. Individual samples were transferred to a sterile 35 or 60 mm Petri dish. The epidermis layer with hair, if present, was removed using sterile forceps and scalpel, leaving only the dermis layer. Fatty tissue and fat globules were also removed. The tissue sample was rinsed with transport media. A 5–10 mm^2^ piece of the cleaned sample was disassociated either with forceps and scalpel, or wide-bore syringe and needle, and transferred to a fresh 35 mm Petri dish. The tissue was covered with collagenase solution and placed in a 37°C incubator for at least 30 min. After removing the collagenase with a sterile Pasteur pipette, the tissue was covered with Trypsin EDTA/Hank’s balanced salt solution and incubated at 37°C for a further 10–15 min. The solution containing the cell suspension was gently mixed, transferred to a centrifuge tube and centrifuged at 1800 rpm for 8 min. After removing the supernatant, 6 ml of F10 media was added and the sample gently resuspended. The sample was split between two cell culture flasks and transferred to a 37°C incubator. Fibroblast cultures were propagated in Hams F10 media (Sigma) or GIBCO® AmnioMAX^™^ Basal Medium (450 ml) with GIBCO® AmnioMAX^™^ C100 supplement (75 ml), newborn calf serum (Sigma), l-glutamine and amoxicillin (0.5 ml).

### Derivation of EBV lymphoblastoid cell lines from whole blood

Whole blood taken by venous puncture and collected in ACD-B tubes was shipped on ice. After spinning, lymphocytes were collected and transformed as previously described by Public Health England (https://www.phe-culturecollections.org.uk/services/hgs/ebvtransformation.aspx).

Exponentially growing cell lines were transported in T25 flasks as growing cultures. Lymphoblastoid cell lines were propagated in RPMI1640 media (Sigma) with glutamine (2 mM) and foetal bovine serum added.

### Site-directed mutagenesis

Site-directed mutagenesis was performed using QuikChange® Multi Site-Directed Kit (Agilent Technologies) according to the manufacturer’s protocol. The mutagenic primer (5′-GAGTAAAGAGGTGCGcGAGCATCTGCTCC-3′) was designed according to guidance in the QuikChange® manual and was used to introduce a valine to methionine mutation at position 231 of human WIPI2b in GFP pEGFP-C1-WIPI2b.

### Transfection

HEK293A cells were transfected with the plasmids indicated in figure legends using Lipofectamine® 2000 (Life Technologies). Cells were plated to be 80% confluent on the day of transfection. Transfection mixture was made up of 1 µg plasmid DNA and 6 µl Lipofectamine® 2000 in 1 ml of Opti-MEM^™^ (Life Technologies).

### Immunoprecipitation

Overexpressed GFP-tagged proteins were immunoprecipitated using GFP-TRAP beads (ChromoTek). Transfected cells were washed with PBS and then lysed in 10 mM Tris HCl pH 7.5, 150 mM NaCl, 0.5 mM EDTA, 0.5% NP-40, 1× complete protease inhibitor (Roche). Lysates were clarified at 13 000 rpm at 4°C, and the lysate was diluted to 0.25% NP-40. The lysate was pre-cleared with blocked agarose bead slurry (ChromoTek) before incubating with GFP-TRAP beads (ChromoTek) at 4°C for 1 h. Immunoprecipitates were washed three times with 10 mM Tris HCl pH 7.5, 150 mM NaCl, 0.5 mM EDTA, 1% NP-40, 1× complete protease inhibitor. Protein complexes were eluted with 2× Laemmli sample buffer at 65 °C for 10 min.

For immunoprecipitation of endogenous WIPI2, cells were pretreated with dithiobis(succinimidyl propionate)/Lomat’s reagent (DPS) (Thermo Scientific) at 0.5 mM in PBS for 30 min on ice. Cells were lysed in 1% Triton TNTE (1% Triton^™^ X-100, 20 mM Tris HCl pH 7.5, 150 mM NaCl, 5 mM EDTA) supplemented with 1× protease inhibitor cocktail and the lysate clarified by centrifuging at 13 000 rpm at 4°C. Clarified lysate was incubated with monoclonal WIPI2 antibodies (antibody 2A2), at 4°C overnight before being added to washed protein G beads (Sigma) and incubated for another 2 h at 4 °C. The beads were pelleted (1000 rpm, 4°C for 1 min) and were washed four times in 1% Triton^™^ X-100 TNTE buffer. Protein complexes were eluted and DPS crosslinks were cleaved by boiling the washed bead pellets at 100°C, in 50 µl of 2× Laemmli sample buffer with 50 mM dithiothreitol (DTT), for 10 min.

### SDS-PAGE and western blotting

SDS-PAGE was performed using NuPAGE 4–12% Bis-Tris precast protein gels (Life Technologies). Proteins were transferred to polyvinylidene fluoride (PDVF) membrane (Millipore) using wet transfer. For western blotting, PDVF membranes were blocked with 5% powder milk (w/v) (Marvel) and 0.1 % PBS-tween before incubation with antibodies as indicated in Figures [GFP (Abcam); WIPI2 (2A2, Polson *et al.* 2010)]. HRP-conjugated secondary antibodies (GE Healthcare) and ECL (Amersham, GE Healthcare) were used for protein band visualization. 0.1% PBS-tween was used for washing between antibody incubations.

### Immunofluorescence

Cells were cultured on coverslips for 24 h, and fixed in 3% paraformaldehyde for 20 min. Cells were permeabilized with methanol at room temperature for 5 min and blocked for 1 h in 5% bovine serum albumin (BSA) (Roche), before staining with WIPI2 (2A2, Dundee Cell Products) and LC3 (Abcam) primary antibodies in 1% BSA for 1 h. Cells were washed three times in PBS and stained with secondary antibodies Alexa Fluor® 488 (Thermo) and 555 (Thermo) in 1% BSA for 1 h. Cells were then washed three times in PBS and once in water. WIPI2 and LC3 puncta were analysed by Imaris image analysis software.

### Data availability

The authors are happy to share all of the data reported here.

## Results

### Clinical investigations of a novel developmental phenotype

We identified an extended pedigree with a complex developmental disorder involving mental retardation, speech and language impairment (both receptive and expressive language were affected), cardiac, neurological, thyroid and skeletal abnormalities (Fig. 1). Detailed clinical studies of two affected members of the family (Patients NA and AA), aged 43 and 45, respectively, from two separate branches of the family, were carried out. There were no reported complications of pregnancy in either case, and their birth weights were reported to be normal. Overall, Patient NA appeared to be more affected clinically. One member of the first branch of the family (Patient SG), the brother of Patient NA, died aged 48 years old and was reported to have dysphagia. The affected males from this branch of the family did not marry, but the unaffected brother and the two unaffected sisters are married, producing phenotypically normal offspring.

**Figure 2:**
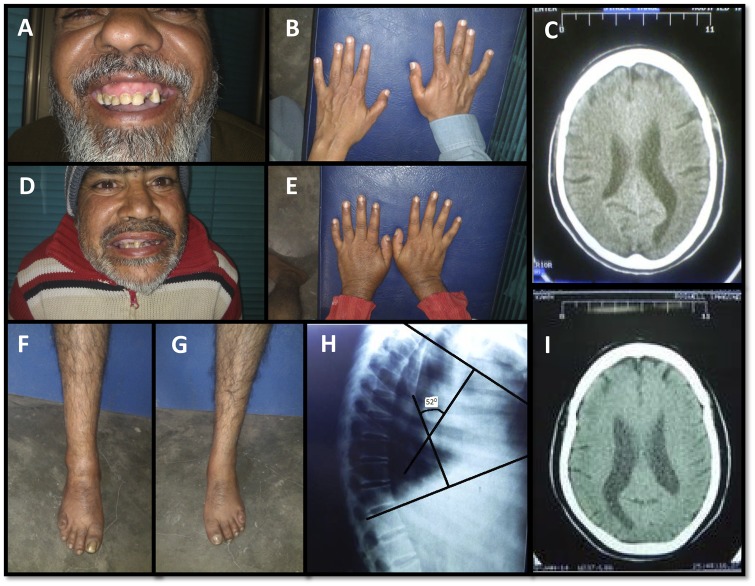
**Skeletal and neurological abnormalities in patients.** (**A**–**C**) Patient AA, aged 43 years, and Patient NA (**D**–**I**), aged 45 years, show a range of clinical abnormalities, including clinodactyly, overlapping of fifth toes, increased thoracic kyphosis, thumb hypoplasia and enlarged ventricles in the brain.

Primarily, the disease manifests as severe mental retardation. The IQ of patients is significantly low and developmental milestones were reported to be significantly delayed. CT scans show mild, but more than expected for the age, global brain volume loss and prominence of the ventricles (Fig. 2). Significant delays were observed in fine/gross motor activities suggestive of a cerebellar dysfunction. Another peculiar finding is short stature in comparison to the rest of the family members and the local population in general. One of the study patients (Patient NA) has mildly increased thoracic kyphosis, mild bilateral thumb hypoplasia, bilateral mild clinodactyly of the little fingers and bilateral overlapping fifth toes (Fig. 2). He also has wide-based gait with short steps. Patient AA has bilateral camptodactyly of the little fingers rather than clinodactyly. Additionally, late onset lumbar vertebrae abnormalities were reported in two members of the extended family (not represented in Fig. 1) that were not age related.

Cardiac abnormalities were also observed on ECG, but did not appear to be clinically relevant. In Patient NA, sinus rhythm P waves appear peaked, with incomplete right bundle branch block and short PR interval and there appear to be delta waves. In Patient AA, sinus rhythm P waves also appear peaked, and interventricular conduction delay is similar to Patient IV-4 but less pronounced, with slurring of R wave in chest leads, but no delta wave. Echocardiography does not suggest any major structural or haemodynamic derangement. Both the study subjects (Patients NA and AA) have subclinical hypothyroidism (normal T3/T4; raised thyroid stimulating hormone).

### Genetic studies to screen for structural variants and point mutations

To investigate a possible involvement of chromosomal aberrations and for the purpose of homozygosity mapping, microarray analysis was performed with a HumanCytoSNP-12 v12.1 chip (Illumina) on nine family members, including four affected individuals from two separate branches of the extended pedigree (Fig. 1). We carried out loss of heterozygosity analysis and identified a single region of homozygosity shared amongst all four affected individuals, a 2.5 Mb region on chromosome 7p22 (positions 3059377–5478971) spanning 31 genes.

**Figure 3:**
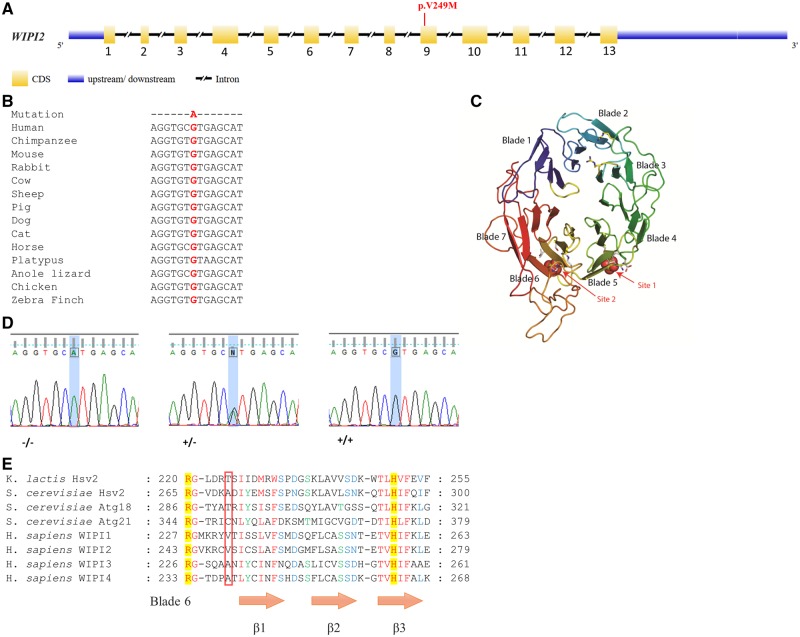
**Genomic structure and mutation in *WIPI2 *gene.** (**A**) The exon-intron structure of the WIPI2 gene is depicted, highlighting the pV249M mutation. (**B**) Phylogenetic analysis demonstrates the cG745A mutation is in a region that is highly conserved across multiple species. (**C**) Model of WIPI2 (Wilson *et al.*, 2014) based on the structure of Hsv2 (Baskaran *et al.*, 2012). WIPI2 is a 7-bladed propeller protein capable of interacting with PI(3)P and PI(3,5)P2 at sites 1 and 2. The WIPI2 pV249M mutation resides within site 2 in blade 6. (**D**) Sanger sequencing confirms the cG745A mutation correctly segregates with the phenotype in 10 family members (Fig. 1). Representative sequence traces from affected (−/−), unaffected (+/−) and control (+/+) individuals are presented, corresponding to genotypes AA, AG and GG, respectively. (**E**) Sequence alignment of PROPPIN family members. The boxed region highlights the position of the pV249M residue and its comparison across PROPPIN family members. Figure adapted from Baskaran *et al. *(2012). Residues involved in binding PI(3)P and PI(3,5)P2, including R243, which is an essential requirement for binding to occur, are highlighted in yellow.

Exome sequencing was performed on Patients NA and AA. Screening for non-synonymous homozygous or compound heterozygous variants and stop codons within the region of homozygosity on chromosome 7 identified by the SNP array (see ‘Materials and methods’ section), resulted in a single gene. We identified a novel homozygous mutation in exon 9 (c.G745A;pV249M) in the WD-repeat protein interacting with phosphoinositide 2 gene (*WIPI2*), at genomic base position 5265458 (Fig. 3A). The mutation affects multiple isoforms and is predicted to be damaging according to the PolyPhen-2 software, with a maximum score of 1.000. The V249 residue is highly conserved across species (Fig. 3B) and is also present in WIPI1, a member of the *WIPI *gene family (Polson *et al.*, 2011). The mutation is located at the start of blade 6 (Fig. 3C), near the site implicated in PI(3)P and PI(3,5)P2 binding, defined as site 2 (Baskaran *et al.*, 2012; Krick *et al.*, 2012; Watanabe *et al.*, 2012) and is C-terminal to the highly conserved residues involved in PI(3)P binding, including the arginine (R) at position 243 (Fig. 3E).

The mutation, was confirmed by Sanger sequencing (Fig. 3D) in 10 family members from two separate branches of the extended pedigree (Fig. 1). As expected, it segregates correctly with the disease phenotype consistent with an autosomal recessive pattern of inheritance. Ethnically matched controls (*n* = 217) were screened to exclude any possible population-specific common polymorphisms. Furthermore, the mutation is absent in the homozygous state in all publically available genomic databases including exome variant server (http://evs.gs.washington.edu/EVS), ExAC (http://exac.broadinstitute.org/) and Greater Middle East (GME) Variome databases (http://igm.ucsd.edu/gme/index.php). ExAC alone represents data from over 60 000 genomes from different ancestries, including African, European, Latino and Asian with the GME databases representing 2497 exomes. The V249M mutation is listed in ExAC, athough it is found in only one allele of 88 940.

### Effect of V249M mutation on formation of autophagosomes

**Figure 4:**
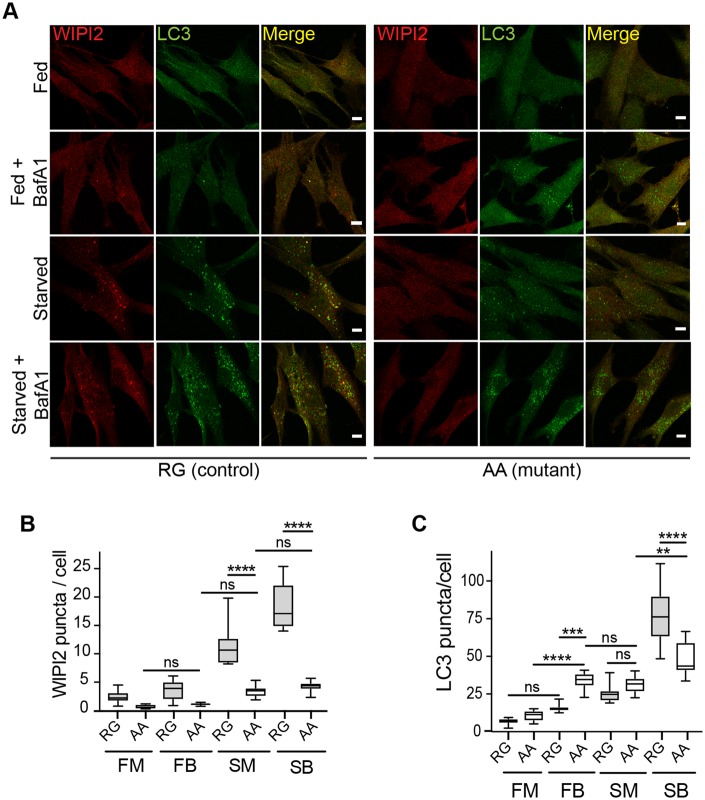
**WIPI2 puncta formation is inhibited in human fibroblasts isolated from patients carrying a WIPI2 mutation.** (**A**) Control (RG) and mutant (AA) human fibroblasts were incubated in either full medium (FM), or treated with 100 nM Bafilomycin A1 in full-nutrient medium (FB), or amino acid starvation medium (SM), or 100 nM Bafilomycin A1 in starvation medium (SB) for 2 h and then fixed. Cells were stained with antibodies against WIPI2 and LC3. Scale bar = 10 μm. (**B** and **C**) WIPI2 and LC3 puncta were quantified in 10 fields per condition. Statistical analysis was performed by one-way ANOVA with Tukey’s post-test. ****P < 0.0001.

WIPI2b is required for formation of autophagosomes and for LC3 lipidation (Dooley *et al.*, 2015). We examined fibroblast cells derived from a patient (Patient AA) and an unaffected family member (Subject RG) to determine if the formation of autophagosomes was altered. WIPI2 and LC3 are cytosolic proteins that are recruited to autophagosomes upon induction of autophagy by amino acid starvation. The recruitment of these proteins is visualized by the formation of punctate structures on the phagophore and the autophagosome (Polson *et al.*, 2010). We incubated fibroblasts from control and patient cells in complete growth medium (fed) or medium lacking amino acids to induce autophagy (starved). As seen in Fig. 4A, LC3 and WIPI2 puncta were formed in starved control cells (Subject RG) as expected. In contrast, in starved patient cells (Patient AA) there appeared to be weakly stained, smaller LC3 puncta; however, there were virtually no WIPI2 puncta detectable. Quantification of the control fed versus control starved cells revealed WIPI2 puncta increased by >10-fold and there was at least a 30–40-fold increase in LC3 puncta (Fig. 4B and C). In contrast, in the patient fibroblasts the number of WIPI2 puncta was significantly decreased to <4-fold (compare Subject RG starved medium versus Patient AA starved medium). The absence of WIPI2 puncta supports the possibility that the VM mutation in WIPI2 alters the association of WIPI2 with PI(3)P and thus the membrane.

Next, we tested levels of autophagy flux in basal and amino acid starvation in the human fibroblasts by inhibiting the lysosomal degradation with the lysosomal inhibitor Bafilomycin A1 (BafA1). This allows the measurement of productive autophagy as a result of autophagosome formation and fusion with the lysosome. In this experimental setting it is important to note only sequestered cargo (LC3, other cytosolic proteins and organelles), which are inside the autophagosome, will be degraded and thus affected (increased) by BafA1 treatment. Therefore, we examined autophagic flux by the addition of 100 nM BafA1 to cells either in complete medium or cells incubated in amino acid free starvation medium and measured LC3 puncta. As seen in Fig. 4C, in patient cells comparing the LC3 puncta in fed medium with the LC3 puncta accumulating in the presence of BafA1 (complete medium basal flux) we see a significant increase. This is not altered by incubation in starvation (compare Patient AA complete medium with starvation medium). However, LC3 puncta were increased upon starvation in the presence of BafA1 (starvation medium) compared to starvation alone, although the total response in Patient AA fibroblasts (starvation medium) was significantly less than the control Subject RG fibroblasts (starvation medium). This suggests the patient fibroblasts have an altered starvation response.

### Effect of V249M mutation on WIPI2 function

We performed functional studies to assess the effect of the mutation in WIPI2 on binding to ATG16L1. Previously, using HEK293A cells expressing GFP-WIPI2b, we showed using GFP pull-downs that ATG16L1 binds to WIPI2b at amino acid residues Arg108 and Arg125. This binding between WIPI2b and ATG16L1 directs lipidation of LC3 to the autophagosome membrane, where it localizes to puncta (Dooley *et al.*, 2014) as shown in Fig. 4. Therefore, to test the effect of the V249M mutation on ATG16L1 binding, we mutated valine in position 231 in GFP-WIPI2b (corresponding to V249 in the canonical isoform1 of WIPI2a) to a methionine to phenocopy the patients’ mutation.

**Figure 5:**
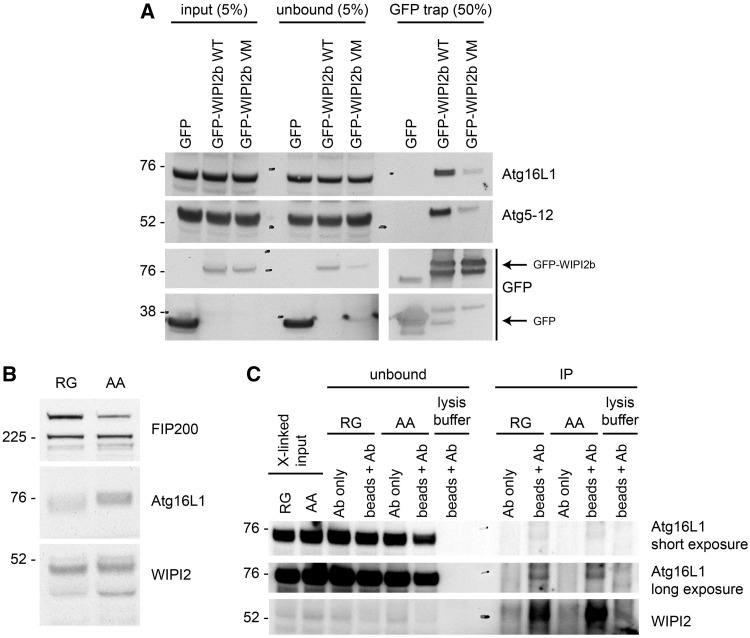
**Analysis of WIPI2 interaction with ATG16L1.** (**A**) GFP-WIPI2b WT or GFP-WIPI2b V231M were transiently expressed in HEK293A cells. The cells were lysed and subjected to immunoprecipitation using GFP-TRAP beads. ATG16L1, ATG12 and GFP (alone or GFP-WIPI2b), in the input, unbound and bound to the GFP-TRAP beads, were detected using the specific antibodies against ATG16L1, ATG12 and GFP. Note data are from same western blot but shown in pieces to aid interpretation. (**B**) Total cell lysate from the unaffected control (RG) and the patient (AA) were analysed for levels of FIP200, ATG16L1 and WIPI2. (**C**) Cell lysates from the control (RG) or the patient (AA) were prepared after the treatment of the respective cell lines with a crosslinker on ice. WIPI2 was immunoprecipitated with an antibody against WIPI2, and the input, unbound and immunoprecipitates were analysed by western blot, using antibodies against ATG16L1 and WIPI2. The WIPI2 immunoprecipitation was done with antibody plus protein A beads (beads + Ab) or antibody alone (Ab).

HEK293A cells were transfected with either GFP-WIPI2b wild-type or GFP-WIPI2b V231M. As seen in Fig. 5A, GFP-WIPI2b V231M showed reduced binding to ATG16L1 compared to the GFP-WIPI2b wild-type. This result suggests that the mutation in V231 reduced the interaction of GFP-WIPI2b with ATG16L1. Given that the V231 mutation in WIPI2b is located near the PI(3)P binding site, and not the ATG16L1 binding site, this result was unexpected.

Therefore, we examined the interaction of endogenous WIPI2 with endogenous ATG16L1 using a crosslinking approach followed by immunoprecipitation, which we developed previously (Dooley *et al.*, 2014). We used lymphoblastoid cell lines from the control (Subject RG) or the affected patient (Patient AA). To ensure that the WIPI2 V231M mutant protein was stable in these cells we blotted Subject RG and Patient AA cells with antibodies to WIPI2, ATG16L1 and FIP200. As seen in Fig. 5B, WIPI2 and ATG16L1 proteins appeared to be stably expressed in Patient AA cells compared to Subject RG control cells. The levels of other ATG proteins involved in the association of ATG16L1 to the autophagosome, such as FIP200 (a member of the ULK1 complex), were unchanged in the patient cells compared to control cells.

Next, following treatment of the cells with crosslinkers at 4°C and lysis, we used an antibody to WIPI2, which recognizes four isoforms of WIPI2(a–d), to immunoprecipitate the crosslinked complex. As shown in Fig. 5C, we were able to immunoprecipitate endogenous ATG16L1 with endogenous WIPI2, but there was no difference in the interaction in Patient AA control cells compared to Subject RG patient cells.

The result in Fig. 5C contrasts with the GFP-WIPI2b V231M mutant in Fig. 5A where the V231M mutant is unable to bind ATG16L1. It may be that the V to M mutation at position 231 causes a conformational change in the GFP-WIPI2b, which reduces ATG16L1 binding. Alternatively, the V231M mutation may cause a reduction in the affinity of WIPI2b to ATG16L1, which is revealed upon overexpression of GFP-WIPI2b V231M, but not under endogenous conditions, in particular because we cannot identify the WIPI2b isoform in endogenous immunoprecipiation. The transient overexpression of GFP-WIPI2b wild-type and/or GFP-WIPI2b V231M mutant upon transfection will bind the ATG16L1 existing in the cell, possibly displacing the endogenous protein, and this binding or competition may be weaker upon mutation of V231M. In contrast, in the control and patient cells it may be that the endogenous WIPI2 proteins are present in complexes, for example with ATG16L1, formed over hours or days. The V to M mutant may have a weaker binding affinity but once bound to ATG16L1 appears to be similar to the normal WIPI2 protein.

## Discussion

We identified a mutation in the major *WIPI2 *gene (pV249M) in an extended family with a complex developmental disorder. This mutation resides in the flexible loop between blades 5 and 6, crucially, adjacent to the previously reported PI(3)P and PI(3,5)P2 binding site (site 2) in blade 6 of the propeller (Baskaran *et al.*, 2012; Krick *et al.*, 2012; Watanabe *et al.*, 2012). The key residues previously described in this interaction, including the highly conserved residue R243, flank the mutation. Site directed mutagenesis of R243, abolishes both PI(3)P and PI(3,5)P2 binding (Baskaran *et al.*, 2012; Krick *et al.*, 2012; Watanabe *et al.*, 2012). The ATG16L1 binding site of WIPI2b is located in the basic cleft between the WD propeller blades 2 and 3 specifically defined by arginine residues R108 and R125, which bind an acidic region of ATG16L1, seemingly at a distance from the mutation in blade 6, but the C-terminal domain of WIPI2b is also thought to be involved in modulating the binding (Dooley *et al.*, 2014).

Four members of WIPI family of proteins (WIPI1–WIPI4) have been described. Classified as seven- bladed β-propeller proteins that bind phosphatidylinositols (PROPPINs) (Dove *et al.*, 2004), they bind specifically phosphatidylinositol 3-phosphate PI(3)P and phosphatidylinositol 3,5-bisphosphate [PI(3,5)P2] and appear to have overlapping roles (Jeffries *et al.*, 2004; Proikas-Cezanne *et al.*, 2004; Polson *et al.* 2010; Dooley *et al.*, 2014; Bakula *et al.*, 2017). WIPI1, WIPI2 and WIPI4 are capable of interacting with each other and share a common interacting partner, NudC (Bakula *et al.*, 2017), although WIPI3 and WIPI4 appear to have a role in pathways both upstream of PI(3)P production but also potentially downstream of LC3 lipidation (Bakula *et al.*, 2017) with WIPI4 potentially involved in the recruitment of ATG2A and B downstream to WIPI2 (Velikkakath *et al.*, 2012).

WIPI2 shares the highest homology with WIPI1 and the V249 residue mutated in our patients is conserved in WIPI1 (Fig. 3E). Whilst WIPI1 plays a supporting role in facilitating the recruitment of ATG16L1 complex for LC3 lipidation by WIPI2 and can associate with ATG16L1, although to a lesser extent than WIPI2 (Bakula *et al.*, 2017), WIPI2 plays a role in localization of WIPI1 to nascent autophagosomes (Bakula *et al.*, 2017).

WIPI2 itself exists as five isoforms (a–e), four of which are characterized. WIPI2b and d are recruited to the membrane upon PI(3)P production by the class III PI3 kinase complex containing Beclin 1 and ATG14 (Mauthe *et al.*, 2011; Dooley *et al.*, 2014). The role of WIPI2b in autophagy begins with recruitment of WIPI2b to the nascent autophagosome in response to PI(3)P production at the endoplasmic reticulum. Production of PI(3)P is initiated by upstream proteins, including VPS34. Recruitment of WIPI2b is followed by the recruitment of the ATG12–5–16L1 complex, to enable conjugation of LC3 conjugation to membrane phosphatidylethanolamine (PE) and lipidation (reviewed by Müller and Proikas-Cezanne, 2015). Our experiments suggest binding of WIPI2b to ATG16L1 (and ATG12-ATG5) might be altered in patient cells studied here. In addition, the patient cells have virtually no WIPI2 puncta, suggesting the recruitment of WIPI2 to the phagophore and autophagosome is altered. We also observe an alteration of LC3 puncta intensity and size and an altered response to starvation stimuli. Basal flux, which may be more relevant for the patient phenotype, appears to be increased but the consequences of this has not been explored.

*De novo *mutations in the *WIPI4 *gene have been identified in five patients with SENDA (static encephalopathy of childhood with neurodegeneration in adulthood), an X-linked neurodegenerative disorder associated with childhood psychomotor retardation, sudden onset progressive dystonia-parkinsonism and dementia, plus iron deposition in the globus pallidus and substantia nigra (Saitsu *et al.*, 2013; Seibler *et al.*, 2018). In addition to the severe psychomotor developmental delay from infancy and severe intellectual disability, the patients become bedridden within a few years of the cognitive decline. The phenotype observed in our patients is associated with less severe neurological symptoms, considering the patients described by Saitsu *et al.* (2013) were mostly bedridden or in a wheelchair. However, the mutations in *WIPI4 *mainly involve protein truncations as well as in-frame and frame-shift insertions, resulting in severely reduced protein expression and appear to be lethal in males. By contrast, we identified a missense mutation and levels of WIPI2 protein expression do not appear to be significantly altered in our patients.

In addition to what we have described, additional clinical signs have been observed in our patients, including signs of ageing, dental enamel eruptions, cataracts and rough and brittle hair. Both of the patients we assessed are reported to have bruxism (teeth grinding), which appears to have caused severe deformities of the teeth. This has previously been reported to occur with lesions of the basal ganglia (Tan *et al.*, 2004). Interestingly, there is a family history of tuberculosis, which has also been linked to autophagy (Castillo *et al.*, 2012; Kimmey *et al.*, 2015). Linking these broader clinical observations with the WIPI2 pathway in the cell, should add to the growing evidence linking autophagy with both normal development and disease processes. Untangling WIPI2 from the overlapping roles of other WIPI family members will no doubt further illuminate this pathway. In addition, alternate pathways involving other proteins including FIP200, which can bind, and possibly recruit, the ATG12–5–16L1 complex to sites of autophagosome as well as the PI(5)P pathway that is independent of PI(3)P (Gammoh *et al.*, 2013; Vicinanza *et al.*, 2015; Vicinanza and Rubinsztein, 2016) might also play an important role.

The precise mechanisms of autophagy are complex and presently unclear. The serendipitous identification of a naturally occurring mutation in *WIPI2 *(within the critical PI(3)P/PI(3,5)P2 binding domain) that leads to fewer autophagosomes in cells derived from patients, should shed further light on the complex mechanisms leading to the mature autophagosome.

## Web resources

Exome Variant Server. NHLBI Exome sequencing Project (ESP). http://evs.gs.washington.edu/EVS/ 2012.

## Supplementary Material

Supplementary Data

## Abbreviations

PI(3)P: phosphatidylinositol 3-phosphate
PI(3,5)P2: phosphatidylinositol 3,5-bisphosphate
