# Supplementary material for: A mutation in the major autophagy gene, WIPI2, associated with global developmental abnormalities
Source: Brain. 2019 Apr 10;142(5):1242–54. doi: 10.1093/brain/awz075 (PMC6487338; doi:10.1093/brain/awz075)
Supplement: Supplementary Data [file awz075_supp.zip › awz075-Suppl_data/awz075_Suppl_2.pdf]

SUPPLEMENTARY MATERIAL

We carried out LOH analysis and identified a single region of homozygosity shared among four affected individuals but not the clinically unaffected members of the family. This 2.5 Mb region on chromosome 7p22 (location: 3059377-54789710) spans 31 genes.

We next performed whole exome sequencing on patients NA and AA and combined the data to exclude variants that are not shared. Within the region of homozygosity identified by the SNP array, we screened for protein altering splice variants and exonic variants, and removed variants observed in 1000 Genomes Project and The Exome Aggregation Consortium occurring at a frequency of less than 0.01. Only one variant remained, in WIPI2. This variant was predicted to be damaging based on SIFT, PolyPhen2, MutationTaster and Provean analyses.

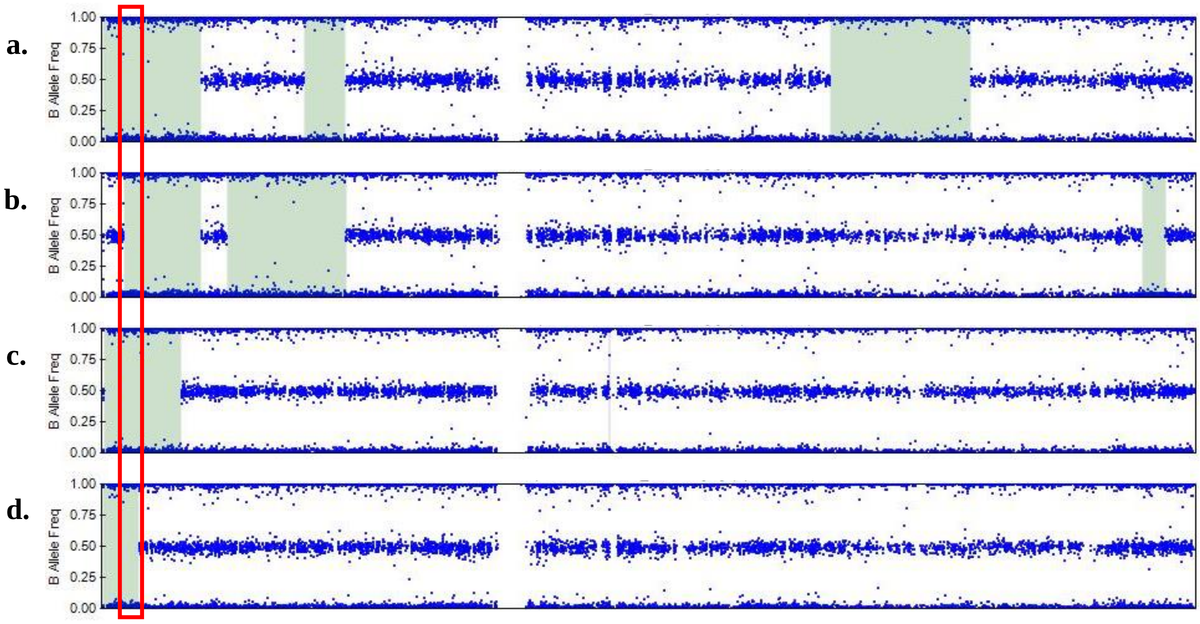

Supplementary Figure S1. B allele frequency (BAF) (blue dots) is defined as the estimated number of B alleles divided by the sum of both alleles at a given SNP location. A BAF of 0 represents the genotype (A/A or A/−), 0.5 represents (A/B) and 1 represents (B/B or B/−). The green areas represent regions of homozygosity. The red box shows the region of homozygosity shared by four affected individuals on chromosome 7. Sample IDs: (a) R01C01-D120423-SG, (b) R01C02-D120429-NA, (c) R05C01-D120427-FAS, (d) R06C01-D120428-AAS.
