# Supplementary material for: A mutation in the major autophagy gene, WIPI2, associated with global developmental abnormalities
Source: Brain. 2019 Apr 10;142(5):1242–54. doi: 10.1093/brain/awz075 (PMC6487338; doi:10.1093/brain/awz075)
Supplement: Supplementary Data [file awz075_supp.zip › awz075-Suppl_data/awz075_Suppl_1.pdf]

Supplementary Material

Supplementary Figure 1. Original western blots for Figure 5A. Western blot with A) Atg16L1, B) ATG5, C) GFP (for GFP-WIPI2) and D) GFP antibodies.

Supplementary Figure 2. Original western blots for Figure 5B. Western blot with A) FIP200, B) ATG16L1, and C) WIPI2 antibodies.

Supplementary Figure 3. Original western blots for Figure 5C. Western blot with A) Atg16L1 (short exposure) B) ATG16L1 (long exposure), and C) WIPI2 antibodies.

A

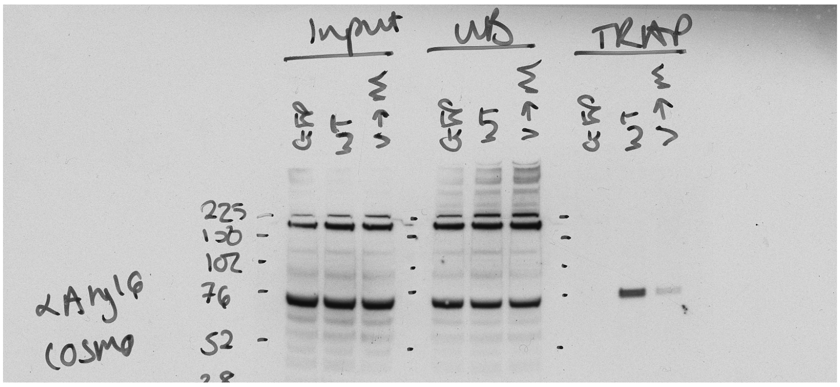

B

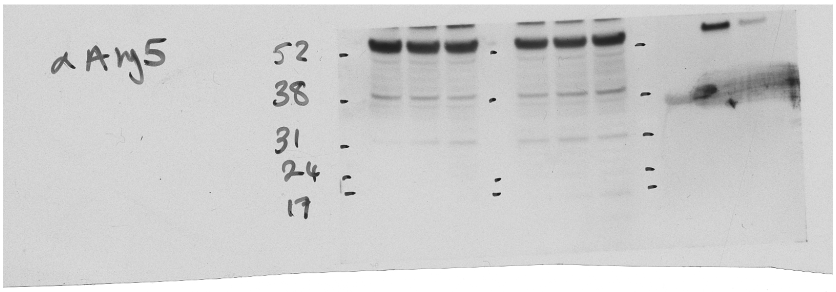

C

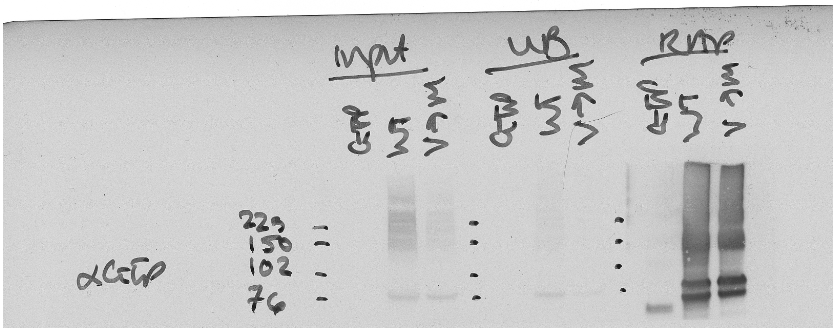

D

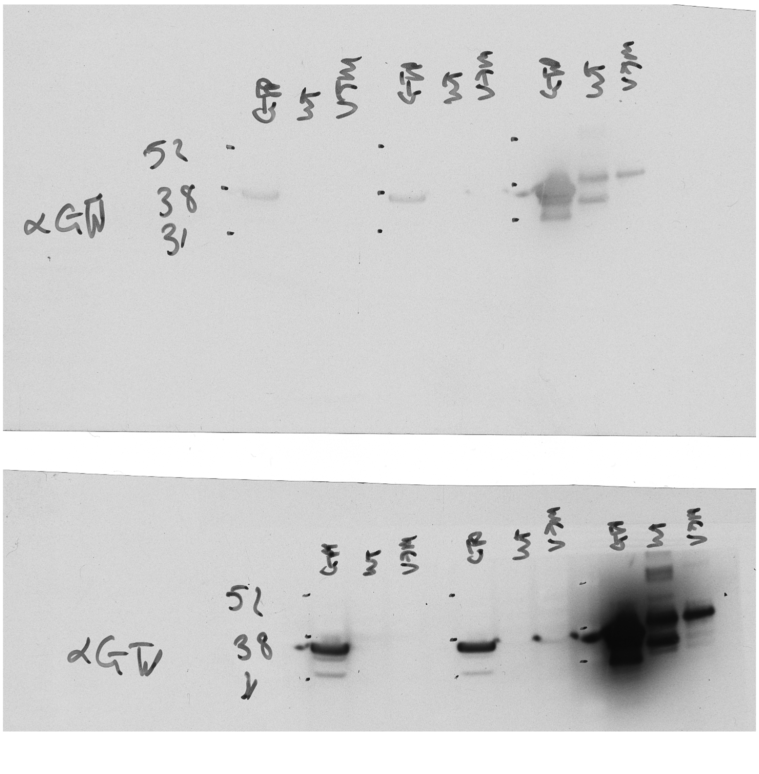

Supplementary Figure 1

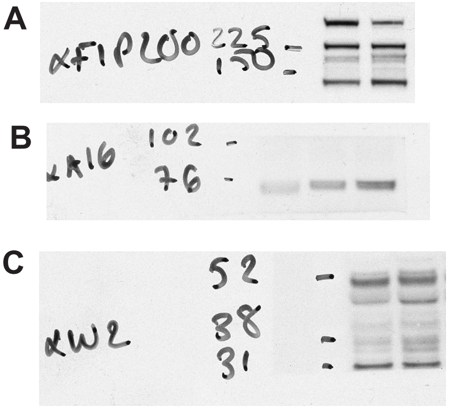

Supplementary Figure 2

A

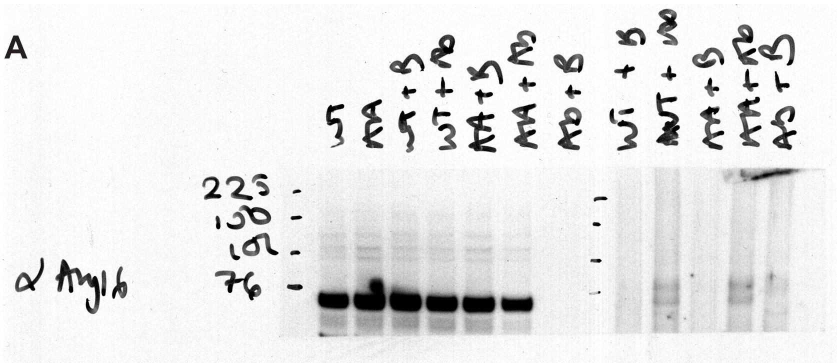

B

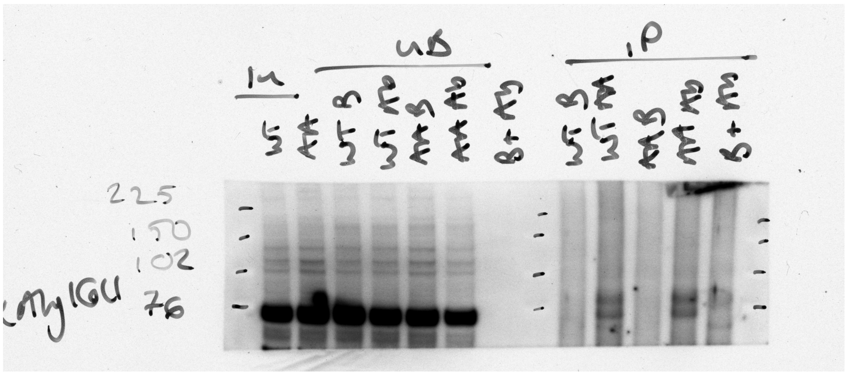

C

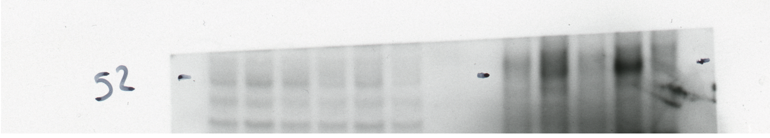

Supplementary Figure 3
